# Antibodies Against Glycolipids Enhance Antifungal Activity of Macrophages and Reduce Fungal Burden After Infection with *Paracoccidioides brasiliensis*

**DOI:** 10.3389/fmicb.2016.00074

**Published:** 2016-02-03

**Authors:** Renata A. Bueno, Luciana Thomaz, Julian E. Muñoz, Cássia J. da Silva, Joshua D. Nosanchuk, Márcia R. Pinto, Luiz R. Travassos, Carlos P. Taborda

**Affiliations:** ^1^Department of Microbiology, Institute of Biomedical Sciences, University of São PauloSão Paulo, Brazil; ^2^Laboratory of Medical Mycology IMTSP- LIM53, University of São PauloSão Paulo, Brazil; ^3^Department of Medicine, Albert Einstein College of Medicine, New YorkNY, USA; ^4^Department of Microbiology and Immunology, Albert Einstein College of Medicine, New YorkNY, USA; ^5^Fluminense Federal UniversityNiterói, Brazil; ^6^Department of Microbiology, Immunology and Parasitology, Federal University of São PauloSão Paulo, Brazil

**Keywords:** *Paracoccidioides brasiliensis*, paracoccidioidomycosis, glycosphingolipids, polyclonal antibodies, nitric oxide

## Abstract

Paracoccidioidomycosis is a fungal disease endemic in Latin America. Polyclonal antibodies to acidic glycosphingolipids (GSLs) from *Paracoccidioides brasiliensis* opsonized yeast forms *in vitro* increasing phagocytosis and reduced the fungal burden of infected animals. Antibodies to GSL were active in both prophylactic and therapeutic protocols using a murine intratracheal infection model. Pathological examination of the lungs of animals treated with antibodies to GSL showed well-organized granulomas and minimally damaged parenchyma compared to the untreated control. Murine peritoneal macrophages activated by IFN-γ and incubated with antibodies against acidic GSLs more effectively phagocytosed and killed *P. brasiliensis* yeast cells as well as produced more nitric oxide compared to controls. The present work discloses a novel target of protective antibodies against *P. brasiliensis* adding to other well-studied mediators of the immune response to this fungus.

## Introduction

The agents of Paracoccidioidomycosis (PCM) are a complex group of fungi within the Paracoccidioides genus comprised of four distinct phylogenetic lineages known as PS2, PS3, S1, and Pb01-like (Matute et al., 2006; Teixeira et al., 2009). PCM is a systemic granulomatous disease initiated by the inhalation of Paracoccidioides sp. conidia that subsequently transform into yeast forms in the lungs. Paracoccidioides sp. grows in the yeast form at human physiological temperature and in the mycelial form at 25°C (Franco et al., 1993). PCM is endemic in a broad region from Mexico to Argentina, although ∼80% of diagnosed patients are in Brazil. Most patients are rural workers but cases also occur in urban centers, especially those located along routes used by migrant workers (Restrepo, 1985; McEwen et al., 1995). Among the fungal diseases, PCM is the prevalent systemic mycosis in Latin America, with the highest mortality rate among the systemic mycoses in Brazil (Prado et al., 2009).

Previous studies have identified different glucans related to dimorphism in Paracoccidioides brasiliensis (Kanetsuna and Carbonell, 1970; Kanetsuna et al., 1972), antigenic glycoproteins including the major diagnostic antigen gp43 (Puccia et al., 1986), galactomannans (San-Blas et al., 2005), brassicasterol, phospholipids (Pereira et al., 2010; Longo et al., 2013), and glycolipids (Puccia et al., 2011). Glycosphingolipids (GSLs) are important molecules in fungi, involved in adhesion, cell recognition, cell differentiation, signal transduction, and regulation of cell proliferation (Barreto-Bergter et al., 2004, 2011; Nimrichter et al., 2005; Bertini et al., 2007). GSLs from Saccharomyces cerevisiae and other species are structurally different from those of mammals mainly due to the presence of inositolphosphoceramide (IPC) as the core structure (Dickson and Lester, 2002). Acidic and neutral GSLs have both been identified in yeast and mycelium forms of P. brasiliensis (Toledo et al., 1995).

Bertini et al. (2007) used 31 sera from PCM patients to determine their reactivity to acidic GSLs Pb-1 and Pb-2 by ELISA. Only the Pb-1 antigen, which has a Galf residue, was reactive with the PCM patients’ sera. Pb-2, which lacks the Galf residue, was not recognized. The titer of antibodies to Pb-1 increased after the start of antifungal treatment and decreased after 5 months of treatment; thus, the Pb-1 ELISA is a potentially clinically useful test to evaluate patient response to therapy. The authors also investigated the role of GSLs in the differentiation and colony formation of P. brasiliensis, Histoplasma capsulatum, and Sporothrix schenckii using three monoclonal antibodies (mAbs) against fungal GSLs: mAb MEST-1 directed to residues of β-D-galactofuranose linked to mannose, mAb MEST-2 directed to glucosylceramide, and mAb MEST-3 directed to Pb-2. These mAbs exerted a strong inhibitory activity on growth, differentiation and colony formation of these fungi. Experiments, however, using mAb MEST-2, showed no significant inhibition of CFUs or effect in the fungal dimorphism (Toledo et al., 2010). On the other hand, addition of purified human antibodies, directed to GlcCer, inhibited cell budding and growth of Cryptococcus neoformans (Rodrigues et al., 2000).

Therapeutic vaccination with fungal antigens or passive transfer of antibodies can boost the cell immune response and add to the protective effect of chemotherapy, eventually counteracting a relapsing disease and reducing fibrotic sequels. Both the innate immune response and the adaptive immunity are important for the antifungal protective effect (Travassos and Taborda, 2012).

The first evidence of antibody-mediated protection against *P. brasiliensis* was shown with the passive transfer of two murine mAbs against a glycoprotein of 70 kD which is recognized by 96% of sera from PCM patients (de Mattos Grosso et al., 2003). Administration of these mAbs led to a significant reduction in the CFUs and the number and size of granulomas in the lungs of experimentally infected mice. Studies on the effect of mAbs to the major diagnostic antigen gp43 provide additional insights into the role of antibody protection in PCM (Travassos and Taborda, 2012).

Given the potential role of GSLs in the virulence of *P. brasiliensis*, we investigated the effect of anti-acidic GSLs polyclonal antibodies on the infection clearance, using experimental prophylactic and therapeutic models and, we have found that at least to polyclonal antibodies to GSL in both models studied are protective to PCM experimental.

## Materials and Methods

### Fungal Strain

Virulent *P. brasiliensis* Pb18 yeast cells were maintained by weekly passages on solid Sabouraud medium (Gibco) at 37°C and were used after 7–10 days of growth. Before experimental infection, the cultures were grown in Sabouraud Broth at 37°C for 5 days (Buissa-Filho et al., 2008). The fungal cells were washed in phosphate-buffered saline (PBS; pH 7.2) and counted in a hemocytometer. The viability of fungal suspensions was assessed by 0.4% Trypan Blue (Sigma) exclusion staining and was always higher than 90% (Taborda et al., 1998).

### Extraction of GSLs

Crude lipid mixtures were extracted from *P. brasiliensis* yeast cells by homogenization using a mixer, three times with 200 mL of 2-propanol/hexane/water (IHW, 55:20:25, v/v/v, upper phase discarded), and twice with 200 mL of chloroform/methanol (CM, 2:1, v/v). The five extracts were pooled, dried on a rotary evaporator, dialyzed against distilled water, lyophilized, suspended in chloroform/methanol/water (30:60:8, v/v/v). Acidic glycolipids from the crude lipid extract were purified by ion exchange chromatography on DEAE-Sephadex A-25 (GE-Healthcare). The elution of the samples was performed following protocols I and II. In protocol I, GSLs were eluted from DEAE-Sephadex A-25 with five volumes of the following solvents (Carlo Erba): (a) CHCl_3_:CH_3_OH:H_2_O (30:60:8, v/v/v); (b) CH_3_OH; (c) Sodium acetate 0.2% in methanol; (d) sodium acetate 0.6% in methanol. Fractions corresponding to the neutral glycolipids were eluted in the first solvent and the acidic fraction was eluted with the third solvent. In protocol II, the fraction of acidic glycolipids was purified by column chromatography on Silica Gel 60 (Merck) using five solvents: (a) CHCl_3_:CH_3_OH (8:2, v/v); (b) CHCl_3_:CH_3_OH (6:4, v/v); (c) CHCl_3_:CH_3_OH (4:6, v/v); (d) CHCl_3_:CH_3_OH (2:8, v/v), and (e) CH_3_CHOHCH_3_:C_6_H_14_:H_2_O (55:20:25, v/v/v). The purity was checked by high resolution thin layer chromatography (HPTLC; Merck) developed in the solvent CHCl_3_:CH_3_OH:CaCl_2_ 0.02% at 60:40:9 (v/v/v). HPTLC plates were sprayed with 90% acetone in primuline (Sigma) and visualized under ultraviolet light. Compounds were revealed with 0.5% orcinol (Sigma), in 3 M sulfuric acid (H_2_SO_4_; Straus et al., 1993).

### Animal Use and Ethics Statement

BALB/c, 6 to 8-week-old male, mice were bred at the University of São Paulo animal facility under specific pathogen-free conditions. All animals were handled in accordance with good animal practice as defined by the rules of the national animal welfare bodies. The Animal Care and Use Committee of the University of São Paulo approved all *in vivo* testing.

### Polyclonal Antibodies to GSL

Polyclonal antibodies were raised in BALB/c mice by four immunizations with 50 μg of purified GSLs in Incomplete Freund Adjuvant, intraperitoneally. The animals were bled 24 h before the immunizations, to collect the pre-immune serum. ELISA was used to analyze the immune sera. Polyclonal antibodies obtained from the animals were purified by affinity chromatography using a protein-A column, according to the manufacturer’s direction (Thermo Scientific, Netherlands). Protein-A tightly binds IgG2a, IgG2b, and IgG3, while it binds weakly to IgG1 and does not bind IgM. The polyclonal antibodies were dialyzed and concentrated by AMICON system with total concentration being determined by Nanodrop 1000. ELISA was also used to titer anti-GSL antibodies. The control polyclonal serum was generated in the same manner, except that bovine serum albumin (BSA-Sigma) was used as the immunogen.

### Intratracheal Infection of BALB/c Mice

BALB/c mice were inoculated intratracheally (i.t.) with virulent *P. brasiliensis* Pb18. Mice were anesthetized intraperitoneally (i.p.) with 200 μl of a solution containing 80 mg/kg ketamine and 10 mg/kg of xylazine (União Química Farmacêutica, Brazil). For inoculation, the mouse’s neck was hyperextended, the trachea was exposed at the level of the thyroid, and 3 × 10^5^ yeast cells in PBS were injected i.t. using a 26-gage needle. Incisions were sutured with 5-0 silk.

### Protective Effects of Polyclonal Antibodies to Acidic GSL

We studied two protocols: prophylactic and therapeutic. In the prophylactic protocol, mice were treated with 1 mg antibodies to GSL i.p. and then infected 24 h later with *P. brasiliensis*. These mice were euthanized 15 and 30 days after infection. For therapeutic protocols, animals were first infected i.t. and then immunized i.p. 30 days later with 1 mg of polyclonal antibodies to GSL. These mice were euthanized at either 45 or 60 days after intratracheal infection. In both therapeutic and prophylactic protocols, experimental groups included mice that received 1 mg of antibodies to BSA as a control for the polyclonal antibodies.

### Fungal Burden in Organs of Infected Mice

Fungal burdens were determined by CFUs counting. Sections of lungs were removed, weighed, and homogenized in 1ml of PBS. Samples (100 μl) were plated on solid brain heart infusion (BHI) medium supplemented with 4% fetal calf serum (Vitrocell, Brazil), 5% *P. brasiliensis* (strain 192)-spent culture medium, and 100 IU/ml streptomycin-penicillin (Sigma–Aldrich, USA). Petri dishes were incubated at 37°C and colonies were counted after 7 days.

### Histopathology

Sections of murine lungs were fixed in 10% buffered formalin and embedded in paraffin for sectioning. The sections were stained by the Gomori-Grocott method for fungal cells detection and were examined microscopically.

### Cytokine Detection

Sections of excised lungs were homogenized in 2 ml of PBS in the presence of protease inhibitors: benzamidine HCl (4 mM), EDTA disodium salt (1 mM), N-ethylmaleimide (1 mM), and pepstatin (1.5 mM) (Sigma). The supernatants were assayed for IL-4, IL-10, IL-12, TNF-α, and IFN-γ using enzyme-linked immunosorbent assay (ELISA) kits (BD OpTeia, San Diego, CA, USA). The detection limits of the assays were as follows: 7.8 pg/ml for IL-4; 31.25 pg/ml for IL-10, 62.5 pg/ml for IL-12, 15.6 pg/ml for TNF-α, and 7.8 pg/ml for IFN-γ, as previously determined by the manufacturer.

### Phagocytosis Assay

*In vitro* phagocytosis experiments were carried out with the J774.16 macrophage-like cell line. *In vitro* phagocytosis was performed according to our established protocol (Taborda and Casadevall, 2001, 2002) with minor modification. Cells were plated in 96-well tissue culture plates (TPP, Switzerland) at a density of 10^5^ cells per well, stimulated with 50 U/ml recombinant murine IFN-γ (BdBiosciences, USA) and incubated at 37°C overnight. The medium in each well was then replaced with the following additions: (1) acidic glycolipids purified, at 100 and 200 μg/ml; (2) antibodies from mouse serum immunized with GSL, purified by Protein A; (3) polyclonal antibodies to GSL purified by Protein A, at 100 μg/ml, with the concentration determined by ELISA; (4) polyclonal antibodies to BSA, at 100 μg/ml. *P. brasiliensis* cells were added at a ratio of 5:1 macrophages/yeast cells and then incubated at 37°C for 6, 12, and 24 h. The wells were then washed several times with sterile PBS, fixed with cold absolute methanol, and stained with a 1/20 solution of Giemsa (Sigma–Aldrich, USA). Phagocytosed yeasts were counted by light microscopy at 400× magnification. The phagocytic index (PI) is defined as PI = P × F, where *P* is the percentage of macrophages with internalized yeasts and *F* is the average number of yeast cells per macrophage. Experiments were carried out in triplicate and five to eight different fields were counted.

### Antifungal Activity of Macrophages

After incubation of the macrophages, the viability of the *P. brasiliensis* yeast cells was determined by plating supernatants on BHI agar supplemented with 4% FCS and 5% spent culture medium of *P. brasiliensis* strain192. CFUs were counted after 7 days of incubation at 37°C.

### Production of Nitric Oxide

The levels of nitric oxide metabolite (nitrite) in the culture supernatant of macrophages challenged with opsonized yeasts were determined by Griess reaction (Pick and Keisari, 1981). All determinations were performed in triplicate.

### Statistical Analysis

Results were analyzed using GraphPad 5.0 software (GraphPad Inc., San Diego, CA, USA). Statistical comparisons were made by analysis of variance (one-way ANOVA) followed by Turkey–Kramer posttest and student’s *t*-test. All values were reported as means ± standard errors of the mean (SEM). *P*-values of <0.05 indicated statistical significance.

## Results

### Extraction of GSLs

**FIGURE 1:**
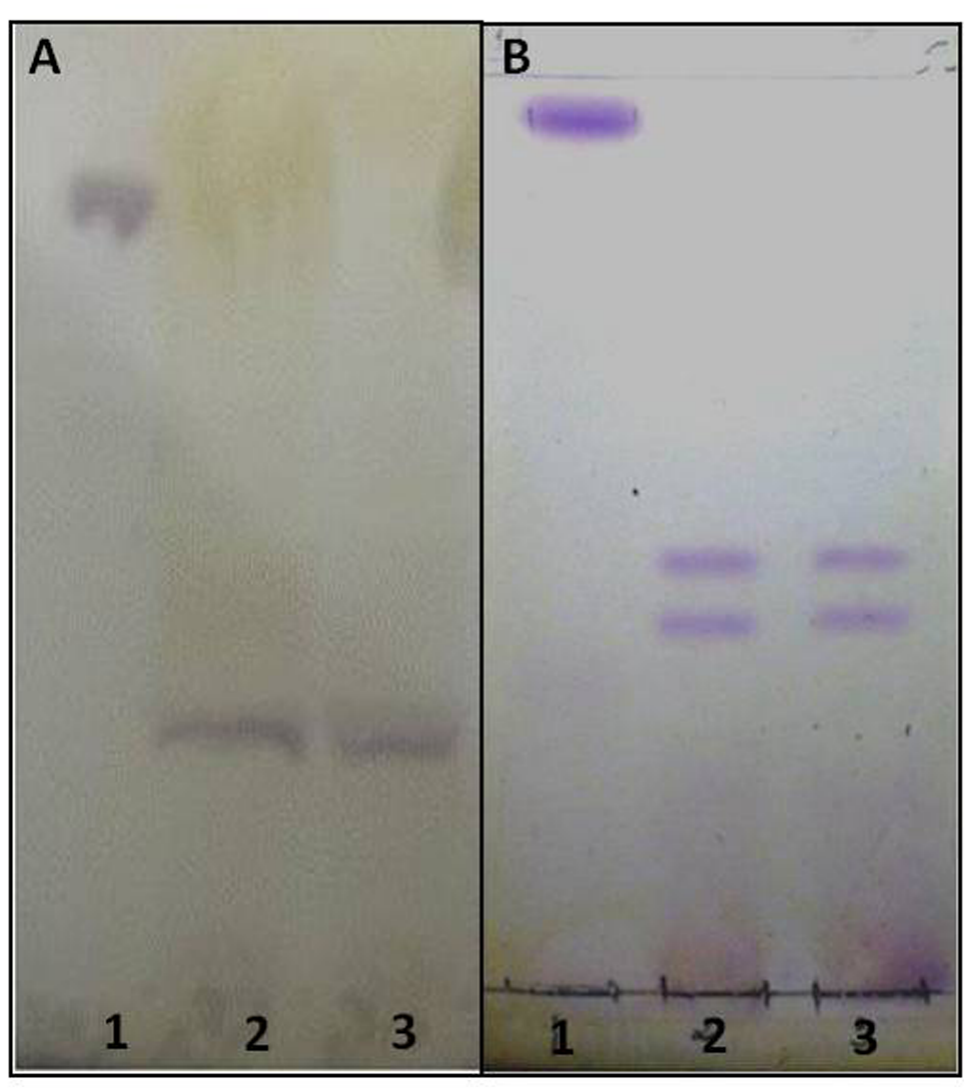
**High resolution thin layer chromatography (HPTLC) of acidic GSLs purified from the crude lipid extract of *Paracoccidioides brasiliensis*.** The solvents used were CHCl_3_:CH_3_OH:CaCl_2_ 0.02% in the proportions 60:40:9 (v/v/v). Revelation with 0.5% orcinol in 3 M sulfuric acid (H_2_SO_4_). **(A)** - (1) Standard CMH, (2) purified acidic GSL (3) purified acidic GSL. **(B)** - (1) Standard CMH, (2) purified acidic GSL Standard Acidic, (3) purified acidic GSL **(B)**. The glycolipids purple stained.

To purify the GSLs, the crude lipid extract was subjected to ion exchange chromatography on DEAE-Sephadex A-25 (protocol I) and A-25 and sequentially in Silica gel 60 (protocol II). The purity was checked by high-resolution chromatography (HPTLC); representative gels of extracted GSLs by protocol I and II are shown in **Figure 1**. For comparison of chromatographic mobility, in the protocol I we used a neutral glycolipid (CMH) standard whereas protocol II used CMH as well as acidic GSL standards.

### Phagocytosis Assay

**FIGURE 2:**
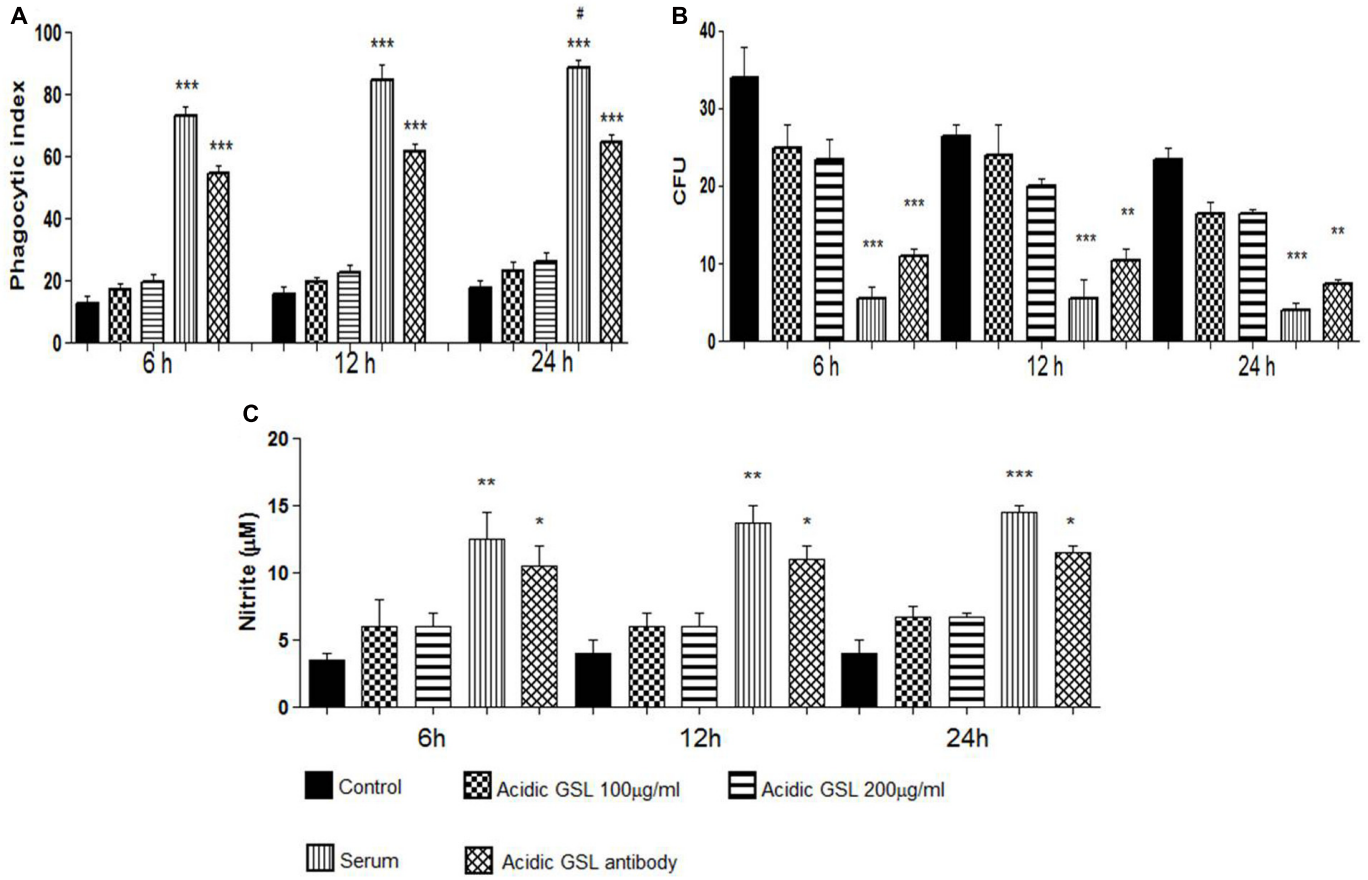
**Experiments *in vitro:***(A)** Phagocytosis of yeast cells by J774.16 cells in the presence of polyclonal antibodies to acidic GSL, polyclonal antibodies to BSA (control), total serum with different polyclonal antibodies and purified acidic GSLs, 100 and 200 μg.** Each bar represents the average of three measurements. Experiments were done in triplicate, and different fields were counted. Significant difference (^∗^*p* < 0.05, ^∗∗^*p* < 0.01, ^∗∗∗^*p* < 0.0001). **(B)** Killing assay using peritoneal macrophages in the phagocytosis assay. Polyclonal antibodies against BSA (control), purified acidic GSL, total serum with different polyclonal antibodies, polyclonal anti-acidic GSL antibody. The columns represent the mean average of three readings. Significant values compared to controls, ^∗∗∗^*p* < 0.0001, ^∗∗^*p* < 0.01. **(C)** Nitric oxide formed in J774.16 cells cultured with *P. brasiliensis* yeasts. Polyclonal antibodies to acidic GSL, polyclonal antibodies to BSA (control), total serum with different polyclonal antibodies, purified acidic GSLs. Nitrite was determined by Griess reagent. Significant difference (^∗^*p* < 0.05, ^∗∗^*p* < 0.01, ^∗∗∗^*p* < 0.0001). ^#^*p* < 0.05 (student’s *t*-test).

The capacity of J774.16 macrophage-like cells for the phagocytosis of *P. brasiliensis* yeast was assessed using different conditions in 6, 12, and 24 h co-cultures with macrophages activated by IFN-γ 24 h before the assays. At all time intervals assessed, incubation in the presence of total serum with polyclonal antibodies against acidic GSL (100 μg/ml) along with the other antibodies present in total serum, significantly enhanced yeast cell uptake by J774.16 (*p* = 0.009), when compared to control groups, cells that received polyclonal antibodies to BSA at 100 μg/ml or only the acidic glycolipids at 100 and 200 μg/ml (**Figure 2A**). It is noteworthy that the total serum contains various kinds of antibodies, not only against GSL, and that these antibodies might induce macrophage-like phagocytosis of *P. brasiliensis* yeasts. However, as can be seen in **Figure 2A**, only the purified GSL antibodies, at all studied times, significantly increased the phagocytosis index, suggesting that only the polyclonal antibody against GSL has the ability to induce phagocytosis in this cell line. The specificity of the polyclonal antibodies was tested by adding glycolipids during phagocytosis, which resulted in a partial inhibition of the process (data not shown).

### Antifungal Activity of Macrophages

The fungicidal effects of J774.16 cells on *P. brasiliensis* was determined in co-cultures using different experimental media. The macrophage-like cells were lysed and the lysates plated onto agar; CFUs were counted after 7 days of incubation at 37°C. It may be observed in **Figure 2B** that the addition of polyclonal antibodies and total serum reduced the viability of the yeast *P. brasiliensis* internalized by the macrophage-like cells, as compared to the control groups. Therefore the polyclonal antibodies to GSL effectively killed *P. brasiliensis*.

### Production of Nitric Oxide

Nitrite levels were detected in culture supernatants of macrophage-yeast co-cultures using a Griess assay. The levels of nitric oxide are intrinsically related to the phagocytosis index. As shown in **Figure 2C** increased nitric oxide was produced in macrophage-like cells incubated with total immune serum and with purified anti-acidic GSL polyclonal antibodies at all incubation times (6, 12, and 24 h), as compared to the control groups. These data are compatible with the results obtained in the phagocytosis and killing assays.

### Fungal Burden in Organs of Infected Mice

**FIGURE 3:**
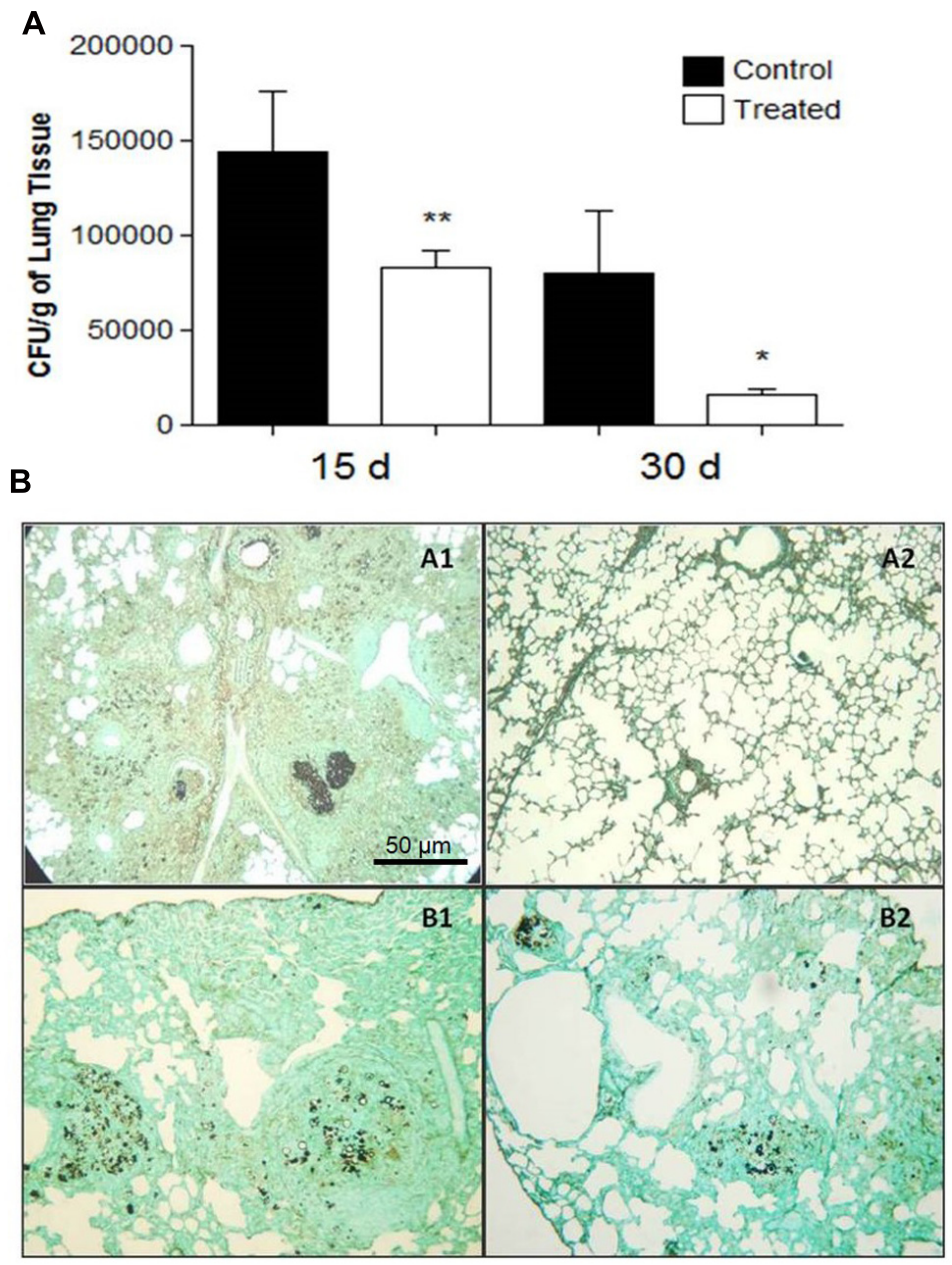
**(A)** Colony forming units (CFU) in the lungs from BALB/c mice that received 1 mg of polyclonal antibodies against acidic glycolipids and 1 mg anti-BSA polyclonal antibodies (control) 24 h before infection with Pb18 and sacrificed after 15 and 30 days (prophylactic protocol). Significant values comparing the lungs of treated and control groups. ^∗^*p* < 0.05, ^∗∗^*p* < 0.01. **(B)** Representative lung sections: Prophylactic protocol. 24 h prior to infection, mice received polyclonal antibodies to acidic GSLs. Histopathological sections of murine lungs 15 (A1, A2) and 30 (B1, B2) days after i.t. infection. (A1, B1) Lungs from control mice; (A2, B2) Histopathological sections after i.t. infection. Photographs of sections were taken at 100× magnification.

Both treatment approaches reduced the fungal burden in animal groups that received polyclonal antibodies against acidic GSL when compared to control groups receiving polyclonal antibodies against BSA. The prophylactic group, immunized 24 h before infection and examined 15 and 30 days afterwards, showed a significant reduction in the fungal burden evaluated by CFUs, *p* = 0.0001 (15 days) and *p* = 0.0129 (30 days), as shown in **Figure 3A**. Moreover the histopathological analyses showed only a few yeasts in the lungs (**Figure 3B**. Slide B2).

**FIGURE 4:**
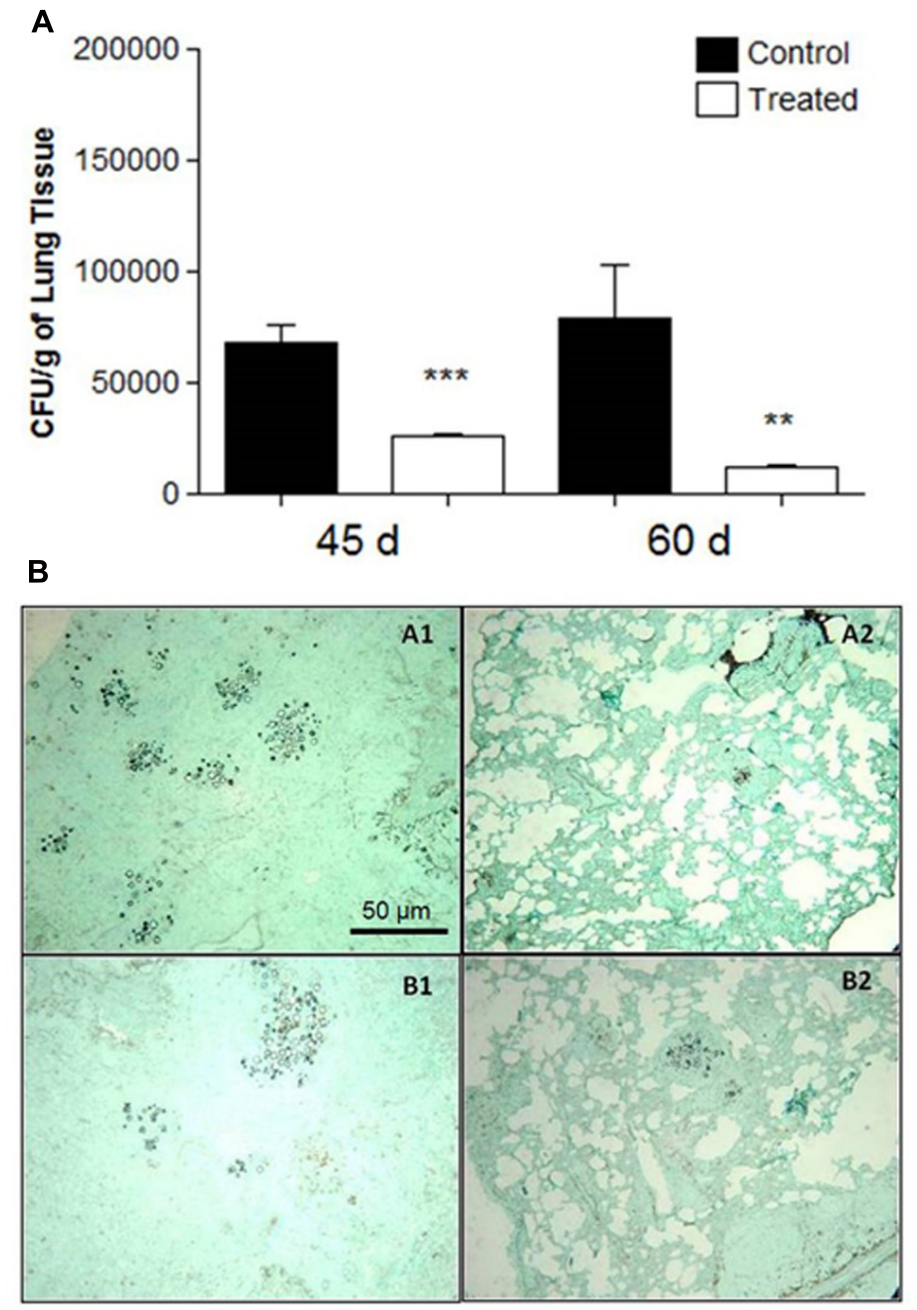
**(A)** Colony forming units (CFU) in the lungs from BALB/c mice that received 1 mg of polyclonal antibodies against acidic glycolipids and 1 mg anti- BSA polyclonal antibodies (control) 30 days after infection with Pb18 and sacrificed after 45 and 60 days (therapeutic protocol). Significant values comparing the lungs of treated and control groups. ^∗∗^*p* < 0.01, ^∗∗∗^*p* < 0.0001. **(B)** Representative lung sections: therapeutic protocol. 3 days after i.t. infection, mice received polyclonal antibodies to acidic GSLs. Controls received polyclonal antibodies to BSA. Representative histopathological sections 45 days after i.t. infection: (A1) Lung from control mouse; (A2) Lung from treated mouse. After 60 days of i.t. infection: (B1) Control; (B2) Treated. Photographs of sections were taken at 100× magnification.

In the therapeutic groups, examined 45 and 60 days after infection with *P. brasiliensis* and with established disease in the animals, CFUs decreased in the lungs, *p =* 0.0001 (45 days) and *p* = 0.006 (60 days) according to **Figure 4A**. No yeasts were detected in the liver and spleen (data not shown).

### Histopathological Analyses

At day 15 after infection, the lungs of the control animals pre-treated with polyclonal antibodies to BSA showed intense infiltrations of inflammatory cells with numerous foci of proliferating fungal cells (**Figure 3B**. Slide A1). In contrast, the mice treated with polyclonal antibodies to acidic GSLs displayed significantly less lung tissue infiltration, few intact yeast cells, and large areas with preserved architecture (**Figure 3B**. Slide A2). After 30 days, the lungs of animals treated with polyclonal antibody to BSA showed compact and loose granulomas with many yeast in the lung tissue (**Figure 3B**. Slide B1). Although it is possible to observe yeasts inside lung granulomas, in animals of the prophylactic group after 30 days, the CFUs did not account for many yeasts, thereby suggesting that they were not viable (**Figure 3B**. Slide B2). The therapeutic protocol also resulted in enhanced protection against tissue damage induced by *P. brasiliensis*. Beneficial results were observed 45 days after infection in the treated group (**Figure 4B**. Slide A2) when compared to the control mice (BSA; **Figure 4B**. Slide A1). As shown in **Figure 4B**. Slides B1 and B2, 60 days after infection, in both protocols, there was reduced fungal burden in the lung tissue. Lung architecture remained largely preserved, well-organized granulomas and a little damage to the lung tissue of mice treated with anti-GSL polyclonal antibodies. The group that received polyclonal antibodies to BSA, however, showed loose granulomas and damage to the lung tissue.

### Cytokine Detection

**FIGURE 5:**
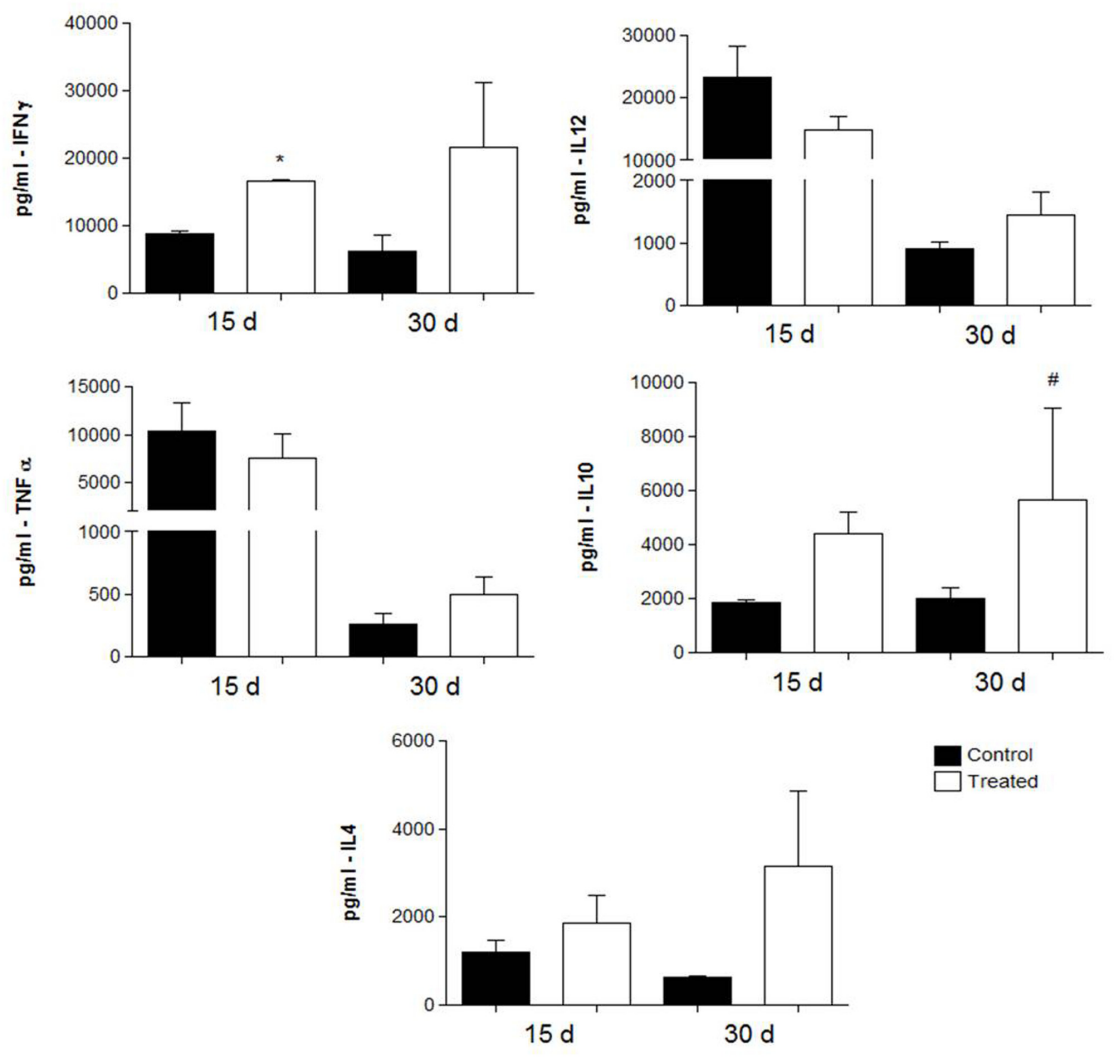
**Cytokine levels in the lungs obtained from BALB/c mice that received either 1 mg of polyclonal antibodies against acidic glycolipids or 1 mg anti-BSA polyclonal antibodies (control) 24 h before infection with Pb18 and sacrificed 15 or 30 days post-infection (prophylactic protocol).** ^∗^*p* < 0.05, ^#^*p* < 0.05 (student’s *t*-test).

**FIGURE 6:**
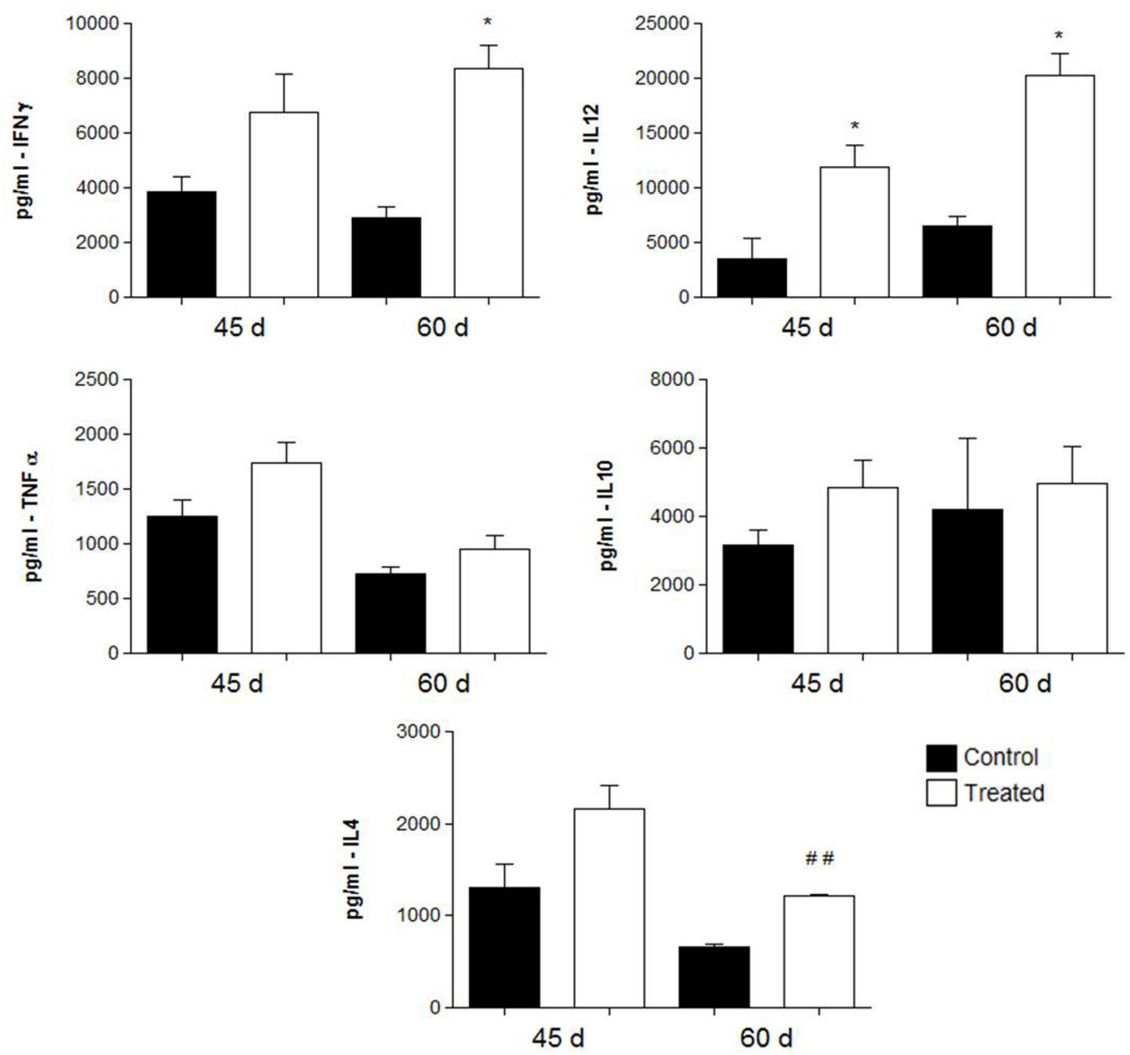
**Cytokine levels in the lungs obtained from BALB/c mice that received either 1 mg of polyclonal antibodies against acidic glycolipids or 1 mg anti- BSA polyclonal antibodies (control) 30 days after infection with Pb18 and sacrificed 45 and 60 days post-infection (therapeutic protocol).** ^∗^*p* < 0.05, ^##^*p* < 0.005 (student’s *t*-test).

Cytokine levels were measured in the lung tissues of i.t. infected mice treated with polyclonal antibodies against either BSA or acidic GSLs. As shown in **Figure 5** (prophylactic group) and **Figure 6** (therapeutic group), in the former, animals immunized with polyclonal antibodies against acidic GSL had higher levels of IFN-γ after 15 days (*p* = 0.0328) of infection compared with mice receiving polyclonal antibodies against BSA. There were no significant differences in the levels of IL-4 and TNF-α. However, IL-10 was statistically different between the groups (*p* = 0.0027).

In the therapeutic protocol, animals immunized with polyclonal antibodies against acidic GSL, showed significantly higher levels of IFN-γ (*p* = 0.0083) in animals sacrificed after 60 days of infection with *P. brasiliensis* and of IL-12 (*p* = 0.0085) after 45 and 60 days of infection.

## Discussion

Studies on antibody immunity against fungal pathogens have revealed that experiments with polyclonal sera may not conclusively validate or disprove the capacity of antibody to effectively modify disease pathogenesis. This quandary arises due to the fact that polyclonal serum may consist of a mixture of protective, irrelevant, or even detrimental antibodies, and the relative quantity of each can dictate whether or not a protective effect is measured (Casadevall, 1995). The polyclonal antibodies also differ in isotype quantity and specificity, which further complicates the assessment of efficacy. Batista et al. (2014) showed that GSLs from *P. brasiliensis* affected the function of human monocytes and dendritic cells, interfering with antigen presentation. Toledo et al. (2010) demonstrated that mAbs against the *P. brasiliensis* glycolipid antigen, had a strong inhibitory activity *in vitro* on differentiation and colony formation of *P. brasiliensis, H. capsulatum*, and *S. schenckii*. Regardless, on the present work, the polyclonal antibodies that we generated to acidic GSLs purified from *P. brasiliensis* demonstrated protective responses in our *in vitro* and *in vivo* infection models. Most significantly, the polyclonal antibodies to acidic GSLs were protective using both the therapeutic and prophylactic protocols in our murine model of intratracheal *P. brasiliensis* infection. The fungal burden was reduced in the lungs of all groups studied, 15, 30, 45, and 60 days after infection. The lung sections from the anti-GSL antibody treated groups exhibited well-organized granulomas and less histopathological damage.

In human PCM, high antibody titers against the major antigens expressed by the yeast *P. brasiliensis* correlate with active disease and the decline in antibody levels is consistent with a response to antifungal therapy and clinical improvement (Restrepo et al., 2008). Patients with clinical forms of the disease usually receive long-term treatment, to allow control of the clinical manifestations of mycosis and to avoid relapses (Del Negro et al., 2000; Shikanai-Yasuda et al., 2006).

The effects of six different IgG2a and IgG2b were evaluated in an i.t. infection of *P. brasiliensis* and the mAbs 19G, 10D (IgG2a), and 3E (IgG2b) significantly reduced lung CFUs. In a phagocytosis assay using peritoneal and alveolar macrophages most anti-gp43 mAbs increased significantly the phagocytosis index, with mAb 3E demonstrating the most impressive effect. The reactivity of mAb 3E was directed to an epitope within the sequence NHVRIPIGYWAV of gp43 shared with β–1,3-glucanases of a few other fungal species (Travassos et al., 2007; Buissa-Filho et al., 2008).

Thomaz et al. (2014) showed that mAbs against *H. capsulatum* hsp60, (4E12 and 7B6, of different isotypes), were biologically active against *P. lutzii*. Both isotypes enhanced *P. lutzii* phagocytosis *in vitro*. Passive administration of the mAbs prior to intratracheal infection of *P. lutzii* in mice significantly reduced the fungal burden in pulmonary tissue.

On testing peritoneal macrophages activated by IFN-γ and incubated with polyclonal antibodies to acidic GSLs, an enhanced fungicidal activity of *P. brasiliensis* yeast forms was observed. Killing internalized yeasts occurred simultaneous with nitric oxide production. The involvement of NO in *P. brasiliensis* killing is inhibited by IL-10 (Moreira et al., 2010). The effectiveness of anti-acidic GSL antibodies *in vivo* reflected the three–fourfold ratio of IFN-γ to IL-10 in the immunized mice. The effect the nitric oxide in experimental cryptococcosis was also related to the modulation of cytokine expression, underscoring the interdependency of cellular and humoral defense mechanisms (Rivera et al., 2002).

The cytokine profiles in both protocols (prophylactic and therapeutic) showed a mixed activation of Th1 and Th2 cytokines, while similar results were observed previously by Buissa-Filho et al. (2008), using a protective mAb against gp43 of *P. brasiliensis*. In our prophylactic protocol, we observed a trend toward increased levels of IFN-γ and IL-10, whereas in the therapeutic protocol IFN-γ IL-4 and IL-12 were more prominent. The role of IFN-γ mediating activated macrophages in PCM was previously documented, in which murine peritoneal macrophages and immortalized cells activated by IFN-γ displayed enhanced fungicidal activity (reviewed by Buissa-Filho et al., 2008 and Taborda et al., 2015). In our present results, polyclonal antibodies against acidic glycolipids enhance IFN-γ in both protocols; however, the best results were observed in the therapeutic protocols. As to the clinical relevance, therapeutic protocols would indeed be an ideal approach for the administration of antibodies for combating PCM.

In the present work, polyclonal antibodies directed to fungal acidic GSLs were shown to exert immune protection against *P. brasiliensis* in an intratracheal infection model.

## Author Contributions

RB and LT – these two authors contributed equally to this work - laboratory experiment. JM, CS, and MP - laboratory experiment - glycolipids purification and analysis. JN and LT - senior researcher - analysis and discussion of results. CT - mentor.

## Conflict of Interest Statement

The authors declare that the research was conducted in the absence of any commercial or financial relationships that could be construed as a potential conflict of interest.
